# Molecular Evidence of *Bartonella* spp. in Rodents: A Study in Pianosa Island, Italy

**DOI:** 10.3390/ani10112070

**Published:** 2020-11-09

**Authors:** Sara Divari, Paola Pregel, Stefania Zanet, Ezio Ferroglio, Francesca Giannini, Frine Eleonora Scaglione, Alex Grinberg, Bartolomeo Biolatti, Enrico Bollo

**Affiliations:** 1 Dipartimento di Scienze Veterinarie, Università degli Studi di Torino, 10095 Grugliasco (TO), Italy; paola.pregel@unito.it (P.P.); stefania.zanet@unito.it (S.Z.); ezio.ferroglio@unito.it (E.F.); frineeleonora.scaglione@unito.it (F.E.S.); bartolomeo.biolatti@unito.it (B.B.); enrico.bollo@unito.it (E.B.); 2 Parco Nazionale Arcipelago Toscano, 57037 Portoferraio (LI), Italy; giannini@islepark.it; 3 School of Veterinary Science, Massey University, 4442 Palmerston North, New Zealand; A.Grinberg@massey.ac.nz

**Keywords:** *Bartonella henselae*, *B. cooperplainsensis*, invasive species, rodents, rats, zoonoses

## Abstract

**Simple Summary:**

Invasive rats and field mice were captured under the project RESTO CON LIFE “Island conservation in Tuscany, restoring habitat not only for birds”, aiming to improve habitats preservation on the Italian islands of Pianosa, Elba, Montecristo, and Giannutri. *Bartonella henselae* DNA was detected in one captured animal. This is the first report of the presence of *B. henselae* DNA in rodents in Italy. *B. henselae* is the causative agent of cat scratch disease, and the detection of this bacterium in rodents might have public health implications.

**Abstract:**

Wild rodents are reservoirs of several *Bartonella* species that cause human bartonellosis. The aim of this study was to assess the presence of *Bartonella* spp. DNA in wild rodents in Pianosa island, Italy. Rats (*Rattus* spp.; *n =* 15) and field mice (*Apodemus* spp.; *n =* 16) were captured and spleen DNA tested for the presence of *Bartonella* spp. by means of an initial screening using a qPCR amplifying a short segment of the 16S-23S rRNA gene intergenic transcribed spacer region (ITS, ~200 bp) followed by conventional PCR amplification of a longer ITS fragment (~600 bp) and of a citrate synthase (*gltA*, ~340 bp) gene segment. A total of 25 spleen DNA samples obtained from 31 rodent carcasses (81%) yielded positive qPCR results. *Bartonella* genus was confirmed by amplicon sequencing. By conventional PCR, eight out of 25 samples (32%) yielded bands on gels consistent with ITS segment, and 6/25 (24%) yielded bands consistent with the *gltA* locus. Amplicon sequencing identified *B. henselae* and *B. coopersplainsensis* in 1/25 (4%), and 4/25 (16%) samples, respectively. Moreover, 5/25 (20%) of *Bartonella* spp. positive samples showed *gltA* sequences with about 97% identity to *B. grahamii*. These results provide support to recently published observations suggesting that *B. henselae* circulates in wild rodent populations.

## 1. Introduction

The genus *Bartonella* includes Gram-negative pleomorphic bacteria infecting endothelial cells and erythrocytes of mammalian hosts [1]. More than 35 species of *Bartonella* have been described [2], and the use of molecular surveys is continuously increasing the list of genetic variants identified within this genus. Some species cause well-documented human diseases, such as *B. quintana*, the agent of trench fever, and *B. henselae*, the cause of the commonly diagnosed cat-scratch disease. Other species are considered emerging pathogens associated with a broad range of clinical conditions, including endocarditis [3].

*Bartonella* transmission to humans is widely believed to be mediated by haematophagous arthropods [4], and an increasing number of surveys indicate rodents as reservoirs of *Bartonella* species [5]. Yet, rodents are not usually listed as reservoirs of *B. henselae* [5,6]. Usually, *B. henselae* is identified in humans and cats and their arthropods; however, two reports of the molecular identification of this species in rodents were previously described. In particular *B. henselae* was identified in *Apodemus sylvaticus* in Denmark [7], and in *Rattus rattus* in New Zealand [8]. Here, a molecular survey of *Bartonella* spp. DNA in wild rats and mice from Pianosa Island in the Tuscan Archipelago National Park of Central Italy is described. To the authors’ best knowledge, this is the first report of the presence of *Bartonella* spp. in rodents from Italy, and the third report, worldwide, of the detection of *B. henselae* in these hosts. 

## 2. Materials and Methods 

This study used carcasses of 15 rats (*Rattus* spp.) and 16 field mice (*Apodemus* spp.) trapped using T-Rex Snap Rat and Sherman Traps placed all along Pianosa Island, Italy, as part of a rodent control program (project LIFE13 NAT/IT/000471—RESTO CON LIFE “Island conservation in Tuscany, restoring habitat not only for birds”). Traps were emptied within 24 h of setting and the carcasses preserved at −20 °C. The carcasses were thawed overnight at room temperature and the spleens collected by incision of the left abdominal wall changing scalpel and gloves between carcasses. The collected spleens were re-frozen at −20 °C until analyzed. 

The spleens were thawed at room temperature and DNA was extracted using kits (GenElute Mammalian Genomic Miniprep Kit, Sigma Aldrich, St. Louis, MO, USA), according to the manufacturer’s instructions. Initial screening for *Bartonella* spp. was performed by means of a quantitative Real-Time PCR (qPCR) designed to amplify a ~200 bp segment of the *Bartonella* spp. 16S-23S rRNA intergenic transcribed spacer (ITS), using previously described primers [9]. Amplification was performed using the CFX Connect^TM^ Real-Time PCR Detection System (BioRad, Hercules, CA, USA), in a PCR mixture consisting of 10 µL of iTaq Universal SYBR^®^ Green Supermix (BioRad, Hercules, CA, USA), 300 nM of each primer, 1 µL of DNA to a final volume of 20 µL with nuclease-free water. The protocol included a 4-min step at 94 °C followed by 45 cycles at 95 °C for 5 s and 60 °C for 20 s. Samples with a quantification cycle (Cq) less than 35 were considered positive, and the amplicons were sent for bidirectional sequencing to a commercial sequencing provider (BMR Genomics, Padova, Italy) using the same primers.

All the samples yielding ITS sequences consistent with *Bartonella* spp. were further analyzed by conventional PCR (cPCR) at two loci: ~340 bp segment of citrate synthase gene (*gltA*), and ~600 bp- segment of ITS locus. cPCRs were performed using previously described primers [9,10,11], using 10 µL of a master mix (HotStarTaq; Qiagen, Hilden, Germany), 400 nM of each primer and 2 µL of DNA template. For the 600bp-ITS, cPCR conditions were heating to 95 °C for 15 min, followed by 35 cycles at 95 °C for 30 s, 55 °C for 30 s and 72 °C for 1 min, and a final extension at 72 °C for 5 min. The *gltA* gene fragment was amplified by heating to 95 °C for 15 min, followed by 45 cycles at 95 °C for 5 s and 60 °C for 45 s. PCR products were electrophoresed in 2% agarose gels, stained by GelRed Nucleic Acid Gel Stain (Biotium Inc., Fremont, CA) and visualized under UV. *Bartonella* sp. FG4-1 DNA was included as positive control and nuclease-free water as negative control in each PCR batch testing. Amplicons showing bands on gels consistent in size with the amplified locus were sequenced as above, and the raw sequences edited using Geneious Prime version 2019.0.4 [12] and compared online to the sequences stored in the National Centre for Biotechnology Information of the United States of America using BLAST (https://blast.ncbi.nlm.nih.gov/Blast.cgi). *GltA* and ITS amplicon sequences identified in this study and from GenBank were aligned with the MUSCLE alignment algorithm and phylogenetic relations were estimated using Tamura-Nei as genetic distance model and Neighbor-Joining as tree building method. Bootstrap calculation was carried out with 1000 resamplings with support threshold ≥70%. These analysis were conducted using Geneious Prime software version 2019.0.4 [12]

## 3. Results

The results of the survey are summarized in Table 1. Spleen DNA from 25/31 rodents (81%) yielded Cq values of < 35 by qPCR (200bp-ITS), and the sequences of all the amplicons aligned with a high degree of similarity to sequences of *Bartonella* genus (data not shown). A total of 10/25 (40%) yielded *Bartonella* DNA by means of cPCR. *GltA* amplicons were obtained by cPCR in 6/25 (24%) qPCR-positive samples, and 600bp-ITS amplicons were obtained in 8/25 samples. Four samples yielded amplicons at both loci. 

In particular, in one animal, a 346 bp *gltA* sequence was identical (100%) to the sequence of *Bartonella henselae* strain Houston-I (GenBank accession number CP020742.1). This *gltA* sequence was deposited in the GenBank as accession number MK562498.

**Figure 1:**
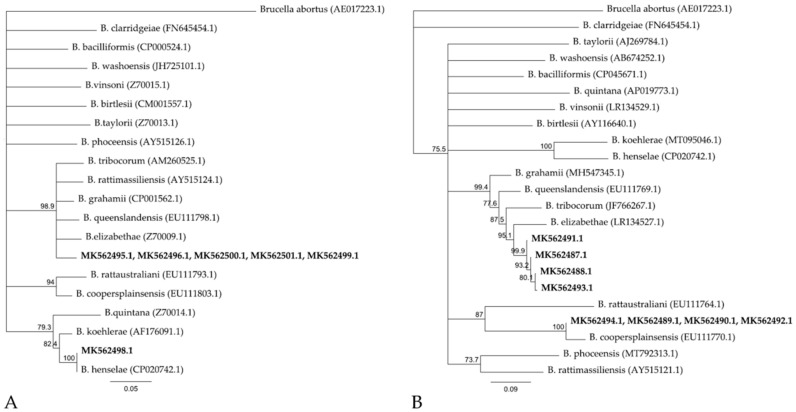
Phylogenetic trees based on the *gltA* (340 bp) (**A**) and ITS (512 bp) (**B**) partial sequences of *Bartonella* identified in the present study (GenBank accession number in bold) and from GenBank (description and GenBank accession number in bracket). The phylogenetic tree was constructed selecting Tamura-Nei as genetic distance and Neighbor-Joining as tree building method. Bootstrap calculation was carried out with 1000 resamplings with support threshold ≥ 70%. Phylogenetic trees were built using Geneious Prime software version 2019.0.4.

The sequences of the other five *gltA* amplicons were identical with each other and shared 96–97% sequence similarity with the *gltA* gene sequence of *B. grahamii* (accession number CP001562.1), and other *Bartonella* spp. (Figure 1). These sequences were deposited in the GenBank under accession numbers MK562495, MK562496, MK562499, MK562500, and MK562501. The ~600 bp- ITS sequences of four samples were 100% similar to the sequence of *B. coopersplainsensis* (accession number EU111770.1) and were deposited in the GenBank as accession numbers MK562489, MK562490, MK562492 and MK562494. The other four ITS sequences were unique and shared 96-97% similarity with the Uncultured *Bartonella* sp. Clone MS_Seville-07021427 (GenBank accession number EU218552.1). These four sequences were deposited under accession numbers MK562487, MK562488, MK562491, MK562493, as Uncultured *Bartonella* sp. 16S-23S ribosomal RNA intergenic spacer, partial sequence. 

*gltA* and ITS sequences of *Bartonella* identified in the present study were compared to related *gltA* and ITS *Bartonella* sequences from GenBank, showing that both *gltA* and ITS novel sequences were closely related to *B. grahamii*, *B. elizabethae*, *B. tribocorum,* and *B. queenslandensis* (Figure 1).

## 4. Discussion

We performed a molecular survey of the occurrence of *Bartonella* DNA in carcasses of wild rodents collected by means of traps in the isle of Pianosa, Italy. Initial screening by qPCR suggested the presence of *Bartonella* DNA in ~80% of the samples, and subsequent cPCR identified *Bartonella* DNA in 40% of the qPCR positive samples. Only four samples were PCR-positive at both loci. These results suggest a greater sensitivity of the qPCR used for screening, as compared with the cPCRs. Many samples were positive at one locus only, highlighting the utility of multiple-locus PCR as a means to increase overall testing sensitivity.

We identified ITS sequences of *B*. *coopersplainsensis* in four rats. *B. coopersplainsensis* was first identified in blood of *Rattus leucopus* in Australia [13], and later in different rodents in Thailand, China, Japan, Taiwan, and New Zealand [8,14,15,16,17]. The zoonotic potential of *B. coopersplainsensis* has yet to be established. 

We found novel *gltA* and ITS sequences in five mice (Table 1). Interestingly, the five positive mice yielded identical *gltA* sequences, excluding the possibility of PCR artifacts. This sequence had not been reported previously in the GenBank and had high degree of identity to *B. grahamii gltA* locus. This *Bartonella* species can cause zoonotic infection, supporting the possible role of wild rodent as reservoir of human infections [3]. Exposure to rats and mice has been associated with rodent-specific *Bartonella* species infections, and infections with *B. elizabethae*, *B. grahamii,* and *B. vinsonii* have been observed [18].

We also identified four novel 600 bp-ITS sequences that showed a high degree of similarity to an Uncultered *Bartonella* sp. [19] which is closely related to *B. elizabethae* and was identified in rodents from Spain. The phylogenetic tree built with *gltA* and ITS cPCR products and compared to other *Bartonella* sequences deposited in GenBank allowed to evidence that the five *Bartonella* spp. identified in *Apodemus* might belong to a novel species closely related to *B. grahamii, B. elizabethae, B. queenslandensis*, and *B. tribocorum,* usually identified in rats and mice, and confirming these mammalian as reservoirs of zoonotic *Bartonella* spp. [18]. Moreover, the phylogenetic analysis confirms that the five sequences of *Bartonella* identified in rats are very close to *B. henselae* and *B. coopersplainsens* species.

Interestingly, DNA of *B. henselae* was identified in one rat, corroborating previous observations in New Zealand [8] and Denmark [7]. This was obtained by amplifying a fragment of the *gltA* locus, which has the largest number of sequences in the GenBank. The cPCR of the 600bp-ITS of this sample did not produce an amplicon, perhaps due to DNA degradation. This is the third report of the presence of *B. henselae* DNA in rodents, worldwide. Hence, rodents might have a greater role in the eco-epidemiology of cat scratch disease than previously thought. The circulation of *B. henselae* in rodents would need to be corroborated by culture [20]. It should be underlined that *Bartonella* culture from wild rodent carcasses is often hindered by the decomposition of the carcasses and the in vitro overgrowth of fast growing post-mortem contaminants. 

## 5. Conclusions

We identified DNA of *B. henselae, B. coopersplainsensis,* and of a potential novel *Bartonella* species in spleen of rodents in the isle of Pianosa, Italy. Rodents may play a previously underestimated reservoir role in the eco-epidemiology of cat scratch disease.

## Figures and Tables

**Table 1:** Detailed results of the *Bartonella* spp. positive samples found in this study. GenBank accession numbers are reported. For each sample, the closest sequence (description, accession number, query cover %, e-value and identity %) are reported in brackets.

Host Genus	qPCR-Positive Samples (n)	DNA Sequences	
		GltA	ITS
*Rattus*	1	MK562498 (*B. henselae;* CP020742.1; 100%; 2.0E-179; 100%)	NA
*Rattus*	4	NA	MK562489; MK562490; MK562492; MK562494 (*B. coopersplainsensis;* EU111770.1; 100%; 0; 100%)
*Apodemus*	4	MK562495 (*B. grahami*; CP001562.1; 99%; 3.0E-158; 97%)	MK562487 (Uncultured *Bartonella* sp.; EU218552.1; 99%; 0; 98%)
		MK562496 (*B. grahamii*; CP001562.1; 100%; 8.0E-159; 96%)	MK562488 (Uncultured *Bartonella* sp.; EU218552.1; 100%; 0; 96%)
		MK562499 (*B. grahamii*; CP001562.1; 99%; 1.0E-156; 97%)	MK562491 (Uncultured *Bartonella* sp.; EU218552.1; 100%; 0; 98%)
		MK562501 (*B. grahamii*; CP001562.1; 99%; 3.00E-163; 97%)	MK562493 (Uncultured *Bartonella* sp.; EU218552.1; 100%; 0; 97%)
*Apodemus*	1	MK562500 (*B. grahamii*; CP001562.1; 99%; 3.0E-158; 97%)	NA
*Rattus*	9	NA	NA
*Apodemus*	6	NA	NA

*gltA*, citrate synthase gene; ITS: 16S-23S rRNA gene intergenic transcribed spacer; NA: not amplified.
